# Pills of Sulphate of Quinine

**Published:** 1853-08

**Authors:** 


*Pills of Sulphate of Quiniae.*—Dr. Pereira directs the pills to be made with conserve of roses, and in the three formulæ given in Pharmacopée Universelle, crumb of bread, honey and conserve of roses, are directed. Dorvault directs in L'Officine, for disulphate, the extract of wormwood; for the acid sulphate, conserve of roses. The pills are not officinal in either of the British Pharmacopœias. In our own officinal directions, in the edition of 1850, gum arabic and honey are prescribed, while in that of 1840 gum arabic and syrup were the excipients.

The use of gum arabic and syrup was abandoned on account of the pills becoming insoluble by keeping. Gum arabic and honey used together are probably less objectionable. The omission of the gum entirely is perhaps an improvement, honey answering the purpose alone. As quinine is now more frequently prescribed in 2, 3 and 5 grain doses than in the one grain dose that used to be given, it is a desideratum to use an excipient which will produce the smallest possible increase of bulk at the same time that it gives a plastic mass.

The following formula is, I think, preferable to those in which gum arabic is employed, as well for the diminutive size as for the increased solubility of the pills:

| | |
|---|---|
| Take of sulphate of quinia | 12 grains. |
| Powdered tragacanth | 1 grain. |

Triturate the powders thorougly together, and add sufficient water to form a plastic mass. Divide this into the required number of pills. Made in this way a three grain pill is not inconveniently large.

The use of simple water as an excipient is, I am told, common in domestic practice in the Southern States. The mass produced in this way possesses too little adhesiveness to render it satisfactory. Tannic acid has been used of late with a view to diminishing the intense bitterness of the quinine, but has not found favor generally, as far as my observation has extended. How far the known insolubility of the tannate of quinia in water should operate against this combination is a question for the therapeutist. Conserve of roses, in addition to its bulk, may be objectionable in this as in some other cases, on the score of containing tannic acid, which it does when made from Rosa gallica.

The following formula I have used for several years with great satisfaction to myself and to those physicians who have prescribed it. It was first suggested by a southern *medical student.*

Take of sulphate of quinia, 20 grains.
Aromatic sulphuric acid, 15 drops.

Drop the acid into the sulphate of quinia on a tile or slab, and triturate with a spatula until it assumes a pillular consistence; then divide into the required number of pills. Made in this way a five grain pill is not inconveniently large.

Although the ingredients when mixed form a fluid, they soon thicken into a paste, and finally become quite solid, and so adhesive as to be readily divided and rolled into pills; care must be taken not to allow the mass to become too dry and brittle before dividing it, as it is liable to do if allowed to remain too long.

In this form a portion of the disulphate being converted into the soluble neutral sulphate, the preparation more nearly resembles the solutions in composition, and is believed to be more rapid and certain in its action.

When it is desired to incorporate other substances in powder with the quinine thus prepared, they should be added to the mass when it is just so soft that, upon their addition, it will immediately assume the proper consistence.

It is not, however, advisable to employ this process when any considerable quantity of other ingredients are prescribed with the quinine, unless a little syrup or honey is also added to prevent the too rapid hardening and consequent crumbling of the mass.—*American Journal of Pharmacy.*

